# Managing the Physiologically Difficult Airway in Critically Ill Adults

**DOI:** 10.1186/s13054-023-04371-3

**Published:** 2023-03-21

**Authors:** Craig Steven Jabaley

**Affiliations:** 1 Department of Anesthesiology, Emory University, Atlanta, GA USA; 2 Emory Critical Care Center, Atlanta, GA USA

## Abstract

This article is one of ten reviews selected from the Annual Update in Intensive Care and Emergency Medicine 2023. Other selected articles can be found online at https://www.biomedcentral.com/collections/annualupdate2023. Further information about the Annual Update in Intensive Care and Emergency Medicine is available from https://link.springer.com/bookseries/8901.

## Introduction

Airway management (i.e., tracheal intubation) in critically ill adults has long been recognized as technically difficult [1]. Myriad patient and environment-specific factors conspire to increase the difficulty of conventional laryngoscopy and other approaches to the airway [2]. However, intubation in critical care settings has benefitted from advances in equipment and techniques originally developed for application in procedural environments: algorithmic approaches, video laryngoscopy, supraglottic airways, and other adjuncts. Their application to critical care settings has helped to increase the accessibility and safety of airway management.

Among this mitigation of technical difficulties, the latent physiologic difficulties of tracheal intubation in critically ill adults have become a new focus. These patients typically require definitive airway management due to organ dysfunction or other manifestations of critical illness, including altered consciousness, respiratory failure, and shock. These and other pathophysiological states increase the risks associated with sedative hypnotic (i.e., induction) agents and their hemodynamic sequelae, apnea during tracheal intubation, and/or transition to positive pressure mechanical ventilation. Therefore, successful airway management in critically ill adults requires planning and execution of strategies that mitigate potential technical and physiological difficulties.

## Adverse Outcomes and Risk Factors

Although clinicians have long been intuitively familiar with the challenges and outcomes of tracheal intubation in critically ill adults, high-quality prospective evidence has emerged to help advance our understanding of global practices and outcomes.

### What Are the Outcomes of Tracheal Intubation in Critically Ill Adults?

Until recently, estimates of adverse event rates have relied on extrapolation from a heterogeneous pool of sometimes retrospective and/or single-center studies [3, 4]. Recognizing these limitations, the literature has suggested that tracheal intubation in critically ill adults is associated with an approximate 30% risk of cardiovascular instability, 20% risk of hypoxemia, and 2–4% risk of cardiac arrest. The Fourth National Audit Project (NAP4) of the Royal College of Anaesthetists examined airway complications in the United Kingdom from September 2008 to August 2009 and was the largest study of airway management complications at the time of its publication [5]. Among other findings, NAP4 identified intensive care units (ICUs) as the setting associated with the most potentially avoidable deaths related to airway management.

In 2021, the long-awaited results of the International Observational Study to Understand the Impact and Best Practices of Airway Management in Critically Ill Patients (INTUBE), a prospective study of tracheal intubation in critically ill adults over 8 consecutive weeks in 197 centers across 29 countries with observations between October 1, 2018 and July 31, 2019, were published [4]. Among the 2964 patients included, the most frequent indications for tracheal intubation were respiratory failure (52.3%), neurological impairment (30.5%), and cardiovascular instability (9.4%). Cardiovascular instability—defined as systolic blood pressure (SBP) < 65 mmHg at least once, SBP < 90 mmHg for > 30 min, new or increased need for vasopressors, and/or need for fluid bolus > 15 ml/kg—was the most common adverse event, occurring in 42.6% of intubations. This was followed by severe hypoxemia—defined as oxygen saturation (SpO2) < 80%—in 9.3%, and cardiac arrest in 3.1%. Of the 1172 occurrences of cardiovascular instability, 1053 (89.9%) involved the need for new or increased vasopressors.

### Which Patients Are at Risk?

Recognizing that the risks associated with airway management may be due to technical and/or physiological difficulty, predicting adverse outcomes requires consideration of both factors. This was exemplified by the MACOCHA score, which includes both anatomical and physiological features to predict difficult intubation and has been incorporated into relevant guidelines (Table 1) [6, 7]. Although the MACOCHA score has been validated, it was not associated with adverse events in the INTUBE study when dichotomized into < 3 or ≥ 3 [4]. In multivariate analysis, factors associated with adverse events included age, history of heart failure, history of hematologic malignancy, cardiovascular instability as an indication for intubation, and other features of hemodynamic compromise.

**Table 1:** MACOCHA score variables [6]

Factor	Points
Mallampati III or IV	5
Obstructive sleep apnea	2
Reduced cervical spine mobility	1
Mouth opening < 3 cm	1
Coma	1
SpO2 < 80%	1
Non-anesthesiologist	1

The MACOCHA score includes seven factors: Mallampati score, sleep apnea, cervical spine mobility, mouth opening, coma, hypoxemia, and non-anesthesiologist

Risk factors for cardiovascular collapse (e.g., cardiac arrest) have also been explored. In a secondary analysis of the cohort used to derive and validate the MACOCHA score, Perbet et al. identified advanced age and more severe critical illness as risk factors for cardiovascular collapse [8]. Subsequently, De Jong et al. identified hypotension, hypoxemia, lack of pre-oxygenation, obesity, and age > 75 years as relevant risk factors for cardiac arrest [9]. Halliday et al. identified hypotension, the need for vasopressors prior to intubation, age, and cirrhosis as the top four risk factors in a secondary analysis of trial data [10–13]. Right ventricular (RV) dysfunction is also an increasingly appreciated risk factor as perturbations in gas exchange, acid/base status, and intrathoracic pressure may all lead to cardiovascular collapse [14, 15]. In a secondary analysis of INTUBE, the risk factors for cardiovascular instability included age, lower blood pressure, lower oxygen saturation, and propofol administration [16].

## Hemodynamic Optimization

Given that cardiovascular instability is the primary risk of tracheal intubation in critically ill adults in the contemporary era, its prevention and management is a natural clinical focus. Furthermore, cardiovascular instability, among other adverse peri-intubation events, has been independently associated with ICU mortality [4, 9, 16–19].

### Is There an Optimal Induction Agent?

The optimal induction agent, if any, for tracheal intubation in critically ill adults remains controversial. Clinical experience suggests that any agent has the potential for hemodynamic trespass, highlighting the importance of clinical judgement. However, extrapolated pharmacokinetic modeling from animal studies reveals that some agents (e.g., etomidate) require less dose reduction than others (e.g., propofol) in the presence of shock [20]. Propofol was the induction agent administered most frequently in INTUBE (41.5%), followed by midazolam (36.4%), etomidate (17.8%), and ketamine (14.2%) [4]. Totaling 109.9% of encounters, approximately 10% of patients received more than one induction agent. In the aforementioned secondary analysis of INTUBE, an inverse probability of treatment weighting approach to causal effect inference suggested that propofol administration was the sole variable independently associated with cardiovascular instability or collapse [16]. This finding parallels previous investigations and clinical experience, and etomidate and ketamine have been recommended as first-line induction agents in critically ill adults [7].

Etomidate, anecdotally more so than any other induction agent, continues to prompt spirited debate [21, 22]. Matchett et al. recently reported the results of the Etomidate Versus Ketamine for Emergency Endotracheal Intubation (EvK) trial in which 801 patients were randomized to receive either etomidate or ketamine for emergency tracheal intubation [23]. The resulting Kaplan–Meier curve was divergent so that patients randomized to etomidate had a significantly higher risk of mortality at day 7 and a non-significantly higher risk at day 28. Etomidate advocates inferred non-inferior outcomes from this convergence on day 28 while its detractors inferred risk of avoidable harm.

Among patients who received ketamine in the EvK trial, 25% sustained post-induction cardiovascular collapse versus 17.4% who received etomidate (mean difference 7.6%, 95% CI 2–13). Highlighting the clinical judgement involved in selecting the induction agent and dose, there was substantial heterogeneity in induction agent dose in the EvK trial, which may have influenced outcomes as dosage was not standardized. Ketamine has sympathomimetic properties but has also been found to exert dose-dependent negative inotropy in vitro [24]. Ketamine may be gaining popularity as it represented 68% of induction agents administered in a European bougie trial, but geographically influenced clinical practice patterns seem to also influence induction agent selection as, for example, ketamine represented only 24% of induction agents in a related North American trial [25, 26].

### What Is the Role of Fluids?

While tracheal intubation is one of the most common ICU procedures, fluid administration is among the most common interventions. Recognizing the risk of hypotension due to induction agents and/or transition to positive pressure ventilation, fluid administration prior to intubation has a reasonable physiological rationale. Vasodilation from induction agents may be offset, and venous return to the heart can be increased even amid increased intrathoracic pressure. However, favorable clinical effects have not been borne out in two trials [12, 27]. In PrePARE, the impact of a 500 ml crystalloid bolus on the primary outcome of cardiovascular collapse was examined; there was no significant effect, but there was a suggestion of benefit in patients who received positive pressure during intubation with non-invasive ventilation (NIV) or bag-mask ventilation [12]. This population was specifically studied in a pragmatic follow-up trial enrolling 1067 patients, and again a 500 ml crystalloid bolus did not impact the primary outcome of cardiovascular collapse [27].

### What Is the Role of Vasopressors?

Evidence to inform optimal selection and approach to vasopressor administration is lacking. However, given that cardiovascular instability accompanies a substantial proportion of tracheal intubations, it follows that the immediate availability of vasopressors should be included as part of routine preparation. Whether administered preventively or in response to hypotension, the immediate readiness of these agents guarantees a short time between the development of instability and treatment. Vasopressors have been included and studied as elements of peri-intubation bundles [28], and a trial is underway to compare the efficacy of preemptive vasopressors against a fluid bolus (ClinicalTrials.gov Identifier: NCT05318066).

## Mitigating Hypoxemia

Hypoxemia is the second most common adverse event associated with tracheal intubation in critically ill adults. Maintaining adequate oxygenation between induction and intubation, sometimes called the apneic interval, is a key element of safe airway management. Acute or chronic lung disease coupled with concerns about aspiration serve to limit the efficacy of traditional pre-oxygenation strategies and diminish enthusiasm for certain rescue approaches.

### Are Standard Pre-oxygenation Strategies Adequate?

Conventional pre-oxygenation approaches often do not meaningfully extend the safe apneic interval, particularly in patients with impaired gas exchange at baseline [29]. Secondary analysis of airway management trial data has revealed a nearly linear, proportionate relationship between SpO2 at induction and the lowest SpO2 during tracheal intubation [30]. This was exemplified in a study by Mort et al. in which 34 consecutive critically ill patients were pre-oxygenated prior to tracheal intubation with 100% inspired oxygen fraction (FiO2) through an adult resuscitator bag for 8 min with serial arterial blood gas analysis [31]. From 0 to 4 min, the mean PaO2 increased from approximately 62 mmHg to 84 mmHg, and from 4 to 8 min the mean increase was only 9 mmHg, with a quarter of patients demonstrating a reduction in PaO2, likely due to atelectasis.

### What About Non-Invasive Ventilation?

NIV has been associated with improved oxygenation during tracheal intubation with fewer adverse events compared to conventional pre-oxygenation [32, 33]. Positive pressure likely helps overcome the absorption atelectasis that develops during pre-oxygenation with high FiO2 and unsupported spontaneous breathing. Despite these advantages, INTUBE showed that NIV use was infrequent in clinical practice, although not all patients may require advanced approaches to pre-oxygenation [4]. From a speculative standpoint, there are several potential barriers to wider adoption. One may be the time required to initiate support de novo or other barriers to easy implementation. Another is that the mask interface must be removed prior to airway instrumentation, at which point oxygen delivery is interrupted. Finally, NIV may risk gastric insufflation and aspiration, which will be discussed subsequently.

### What About Apneic Oxygenation?

High flow nasal oxygen offers the advantage of an unobtrusive nasal interface that can be maintained during airway management. Therefore, high flow nasal oxygen is one modality by which to accomplish both pre-oxygenation and apneic oxygenation during airway management. This dual functionality also complicates literature interpretation, as most studies continue high flow nasal oxygen during airway management. Similarly, studies have used different equipment, each with varying maximal flow capabilities (e.g., 15 versus 60 l/min).

**Fig. 1:**
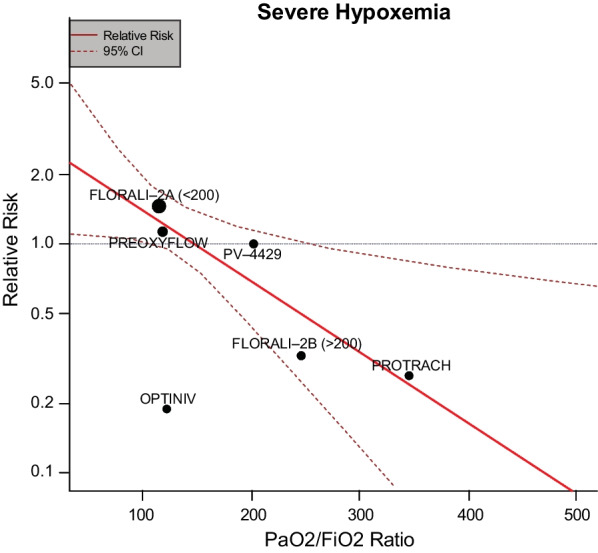
Efficacy of high-flow nasal oxygen for apneic oxygenation relative to the severity of respiratory failure. Relative risk indicates reduction in the incidence of severe hypoxemia, defined as SpO2 < 80%. (Reproduced from [36] under the Creative Commons Attribution 4.0 International License)

Having acknowledged those potential limitations, meta-analyses suggest that pre-oxygenation with high flow nasal oxygen is at least non-inferior to conventional approaches [34–36]. Meta-analyses have also suggested that the efficacy of high flow nasal oxygen is relative to the severity of respiratory failure with limited impact as the severity of respiratory failure increases, for example, as measured by the PaO2/FiO2 (P/F) ratio (Fig. 1) [36]. When comparing the efficacy of high flow nasal oxygen with NIV, therefore, the severity of baseline hypoxemia must be considered. In the FLORALI-2 trial, Frat et al. reported that 24% of patients pre-oxygenated with NIV developed a SpO2 < 80% versus 35% who were pre-oxygenated with high flow nasal oxygen (adjusted odds ratio of 0.56 [95% CI 0.32–0.99]) [37]. The OPTINIV trial explored the combination of high flow nasal oxygen and NIV in 50 patients with a mean P/F ratio of 122 and found that patients receiving the combination intervention maintained a higher SpO2 during intubation than those in the NIV only group [38].

### Should Mask Ventilation Be Avoided?

Critically ill patients are at risk for aspiration and for potentially severe sequelae of aspiration in the presence of acute respiratory failure. Rapid sequence intubation (RSI), which avoids mask ventilation, has long been thought to help minimize the risk of aspiration, although the supporting evidence is limited. Furthermore, RSI itself may confer risks related to induction agent selection and dose and risk of hypoxemia in patients with severe respiratory failure.

The PreVent trial compared bag-mask ventilation versus its avoidance during the interval between induction and tracheal intubation in 401 critically ill adults with a primary outcome of lowest SpO2 [13]. SpO2 was higher in the bag-mask ventilation group (96% vs. 93%), and the incidence of a SpO2 < 80% was lower compared to the control group (10.9% vs. 22.8%). The overall rate of reported aspiration was 3.2%. Although not designed or powered to critically examine safety outcomes, such as aspiration, the PreVent results challenge the dogma that mask ventilation must be strictly avoided and supports clinicians who choose to employ bag-mask ventilation to safely temporize hypoxemia during the apneic interval. Secondary analysis of trial data also suggests that bag-mask ventilation may be associated with higher oxygen saturation during intubation than apneic oxygenation [39].

## First Pass Success

No matter how robust the preparation, time for tracheal intubation may be limited. In the era before video laryngoscopy, multiple attempts at airway management were found to place patients at higher risk for adverse outcomes [40]. In INTUBE, two or more intubation attempts were likewise associated with an increased risk for major adverse events [4]. In particular, the risk of severe hypoxemia increased from approximately 5% with one attempt, to more than 20% with two attempts, and to more than 30% with three attempts.

### Is It Time to Universally Adopt Video Laryngoscopy?

After approximately a century of direct laryngoscopy, video laryngoscopy has increased in popularity, its advocates hailing an emerging standard of care and its detractors bemoaning loss of familiarity with other approaches. Video laryngoscopy is a catchall term for a somewhat heterogeneous group of devices: (1) those with a conventional curved blade profile; (2) those with a hyperangulated blade profile; and (3) those with integral channels for tube passage. Proficiency with one device or category is not necessarily immediately transferrable to another [41]. An updated meta-analysis of 222 video laryngoscopy trials in multiple settings found that video laryngoscopy of any design reduces the probability of failed intubation and complications, with hyperangulated designs performing favorably in those with features of a difficult airway [42]. In ICU video laryngoscopy trials, video laryngoscopy also appears to be associated with improved first pass success [43].

Although an extended discussion about the capabilities and limitations of video laryngoscopy is beyond the scope of this review, three key points are worthy of emphasis. Video laryngoscopy generally gives a superior view of the glottic aperture [42]. However, superior visualization of the airway does not eliminate the need for training and practice to establish expertise. Among trainees using a conventional profile video laryngoscopy device, both the level of training and dedicated video laryngoscopy experience (that is, 15 vs. > 15 intubations) were identified as independent predictors of first pass success when intubating critically ill adults [44]. Using experience from anesthesiology, hyperangulated devices may have a steeper learning curve, with mastery requiring upwards of 70 intubations [45]. Channeled designs inherently aid in tube placement; however, both conventional profile and, more so, hyperangulated devices require a stylet to reliably facilitate endotracheal tube placement. In the absence of a stylet or adequate device-specific expertise, any advantages associated with video laryngoscopy may not materialize, resulting in prolonged airway management and increased risk of adverse events despite superior glottic visualization [46].

### What About Intubation Adjuncts and Checklists?

Despite the growing popularity of video laryngoscopy, direct laryngoscopy remains commonplace worldwide, accounting for 81.5% of intubations included in INTUBE [4]. There also remains international variation in the routine use of endotracheal tube stylets due to their associated risks, which, while uncommon, are potentially severe. The recent STYLETO trial reported a first pass success rate of 78.2% in patients intubated with direct laryngoscopy and a stylet and 71.5% in those intubated without a stylet [25]. Among the 999 included patients, in the stylet group there were two laryngeal injuries, one mediastinal injury, and two esophageal injures, while in the control group there were two laryngeal injuries and one tracheal injury. For clinicians accustomed to using a stylet, the results of the BOUGIE follow-up trial suggest that a tracheal tube introducer (i.e., bougie) may not offer an advantage under usual conditions [26].

Checklists and other similar cognitive aids have been found to increase adherence to complex multistep processes in stressful clinical contexts [47]. ICU intubation checklists that incorporate physiological optimization have been shown to improve outcomes in small studies [28, 48]. Janz et al. conducted the only randomized trial of an intubation checklist and demonstrated no differences in lowest oxygen saturation or blood pressure during intubation; however, that checklist did not include preparatory steps relevant to physiologic optimization [11]. Similarly, a recent meta-analysis of 11 studies, including 3261 patients, found no association between airway checklists and improved clinical outcomes [49]. Although these findings are somewhat discouraging, given the seemingly ever-growing complexity of critical illness and the serious risks posed to patients by airway management in the ICU, further development and assessment of checklists incorporating preparation for physiological difficulties is an important avenue of investigation.

## Is It Time for New Approaches?

Some patients in physiological extremis may not tolerate some or all elements of traditional approaches to emergency airway management, including sedative hypnotic agents, apnea, and positive pressure ventilation. Patients with severe respiratory failure, advanced shock, RV failure, and refractory acidosis are at particularly high risk. In such instances, awake intubation may be considered; however, related techniques may be unfamiliar to some intensivists without practical experience in other contexts (e.g., procedural environments). Training for awake tracheal intubation, awake transition to extracorporeal support, and other such avenues represents a potentially fruitful and important avenue for continued evolution in our management of physiologically challenging scenarios.

## Conclusion

Tracheal intubation in the ICU is a commonplace and short procedure that poses risks to patients and challenges to intensivists. Time and dedication have led to the evolution and refinement of technical approaches to airway management. In this modern era, physiological compromise poses a greater risk to patient safety during airway management in critically ill adults than outright failure of intubation [4]. Therefore, airway management in the ICU has expanded to include preparation for and management of physiologic trespass during tracheal intubation. With this expanded scope comes additional complexity and nuance that require the integration and clinical application of multiple key concepts to each airway management encounter (Table 2).

**Table 2:** Summary points for management of the physiologically difficult airway

**Risks and risk prediction**
Cardiovascular instability, hypoxemia, and cardiac arrest are the most common adverse events associated with tracheal intubation
Risk factors for cardiovascular collapse include age, shock, hypoxemia, advanced critical illness, and propofol administration
**Hemodynamic optimization**
Etomidate and ketamine may impact hemodynamics less than propofol
A crystalloid bolus prior to intubation has not been associated with improved hemodynamics, even in patients receiving positive pressure ventilation
Given the frequency of cardiovascular instability, vasopressors should be readied as part of preparation for tracheal intubation
**Mitigating hypoxemia**
Standard pre-oxygenation strategies are inadequate to safely extend the apneic interval in patients with moderate to severe respiratory failure
Non-invasive ventilation can be used with or without high flow nasal oxygen and is more effective than high flow nasal oxygen alone
While historically avoided, bag-mask ventilation improves oxygenation during airway management and can be employed either preemptively or for rescue
**First pass success**
Multiple attempts at intubation increase the risk of adverse events
Depending on the preferences and expertise of the intubating clinician, video laryngoscopy or direct laryngoscopy with adjuncts may improve first pass success
Checklists improve adherence to complex, multi-step processes and may help prompt preparation for physiologic trespass

## Data Availability

Not applicable.
